# Evaluation of the Relationship Between Sarcopenia and the Geriatric Nutritional Risk Index in Elderly Patients With Type 2 Diabetes Mellitus

**DOI:** 10.1155/ije/7492307

**Published:** 2025-12-04

**Authors:** Aslıhan Calim

**Affiliations:** Department of Internal Medicine, Sisli Etfal Training and Research Hospital, University of Health Sciences, Istanbul, Turkey

**Keywords:** geriatric nutritional risk index, sarcopenia, type 2 diabetes mellitus

## Abstract

**Aim:**

We sought to evaluate the association of the Geriatric Nutritional Risk Index (GNRI), which assesses nutritional status, and sarcopenia among older individuals with type 2 diabetes mellitus.

**Methods:**

We enrolled 292 type 2 diabetes mellitus patients aged 60 years and above in this cross-sectional study. This study took place at Şişli Hamidiye Etfal Training and Research Hospital (Istanbul, Turkey) between April 2024 and December 2024. European Working Group on Sarcopenia in Older People-2 (**EWGSOP2**) **criteria were used to define sarcopenia.** The relationship between sarcopenia and GNRI was investigated by logistic regression models.

**Results:**

The average age was 72 years (range: 60–99). Of the 292 patients, 139 were male and 153 were female. Macrovascular complications and microvascular complications, such as neuropathy and nephropathy, were more common in sarcopenic patients. Low GNRI (< 98) was observed to be more in sarcopenic patients (*p* < 0.001). Multiple logistic regression analysis revealed an association between sarcopenia and neuropathy (*p* = 0.002) and macrovascular complications (*p* = 0.038).

**Conclusions:**

Sarcopenia was more common in elderly type 2 diabetic patients with low GNRI. Our study emphasizes the high rate of malnutrition among sarcopenic patients, with a need for regular screening programs and the determination of elderly subjects requiring nutritional support. GNRI may serve as a screening indicator for the detection of malnutrition and sarcopenia in older diabetic individuals who are hospitalized.

## 1. Introduction

Sarcopenia and malnutrition are among the most important geriatric syndromes. Malnutrition and sarcopenia are closely linked, and both conditions are present in many patients. The frequency of sarcopenia is increasing in individuals with type 2 diabetes mellitus (T2DM) [1]. The increase in the frequency of sarcopenia is associated with conditions, such as frailty, falls, and mortality [2]. Additionally, a relationship has been found between diabetic complications and sarcopenia [2, 3].

Malnutrition among older individuals with T2DM can be influenced by factors, such as age, comorbidities, changes in appetite, mobility limitations, medications, and economic status. Malnutrition can enhance the risk of sarcopenia in elderly diabetic patients [2]. The most commonly used test for screening malnutrition in the elderly is the Mini Nutritional Assessment (MNA) test. The Geriatric Nutritional Risk Index (GNRI) is a diagnostic index proposed for hospitalized elderly individuals, calculated using serum albumin levels, body weight, and height [4]. Previous studies have shown that low GNRI in patients with hemodialysis and cirrhosis is associated with decreases in muscle mass, muscle strength, and walking ability [5, 6].

The exact cause of sarcopenia in patients with diabetes mellitus has not been fully elucidated. In the pathogenesis, insulin resistance, mitochondrial dysfunction, and inflammation are considered responsible [7].

Our study sought to examine the screening of sarcopenia among older individuals with T2DM and its relationship with age, gender, body mass index (BMI), and the GNRI.

## 2. Methodology

A total of 292 patients aged 60 years and above with T2DM, who were admitted to the Internal Medicine Department of the University of Health Sciences, were included in the study. The demographic characteristics, data related to T2DM, and complications of the patients were noted.

The MNA test was used to determine the risk of malnutrition. A hand dynamometer was used to measure handgrip strength, and walking speed was measured using the 4-m walking test. The European Working Group on Sarcopenia in Older People-2 (EWGSOP2) criteria were used to diagnose sarcopenia [8]. According to the revised EWGSOP2 (2018) recommendations, patients were grouped into those with possible sarcopenia with low muscle strength and those without sarcopenia with normal muscle strength.

Muscle strength was assessed by measuring the grip strength of the dominant hand using a Saehan dynamometer. Low muscle strength was characterized as less than 27 kg for men and less than 16 kg for women. A calf circumference measurement of < 31 cm was considered an indicator of reduced muscle mass [8]. In the 4-m walking test, a walking speed of less than 0.8 m/s was defined as slow gait.

Each patient's BMI was determined by dividing their weight in kilograms by their height in meters squared (weight/height^2^).

The following equation was used to calculate GNRI: 14.89 × serum albumin (g/dL) + 41.7 × (body weight/ideal body weight). The Lorentz equations were used to get the formula's optimal body weight [9]. In this study, the cutoff value of 98 was determined a priori, as GNRI < 98 has been consistently used in elderly and diabetic populations to indicate nutritional risk or malnutrition. Accordingly, patients were divided into two groups: those with a low GNRI score (< 98) and those with a high GNRI score (≥ 98). This stratification enabled the evaluation of clinical and metabolic characteristics according to nutritional risk status, consistent with prior studies in elderly and diabetic populations.

Frailty status was assessed using the Frail score. In the Frail questionnaire (0–5), a score of ≥ 3 indicated frail, a score of one to two indicated prefrail, and a score of 0 indicated nonfrail [10, 11]. The nutritional condition among older individuals was evaluated using the MNA test. In this evaluation, scores below 17 indicated malnutrition, scores between 17 and 23 signified a risk of malnutrition, and scores of 24 or higher reflected adequate nutritional condition [10].

Patients with advanced dementia, immobility, bedridden status, T2DM, liver cirrhosis, those undergoing dialysis, and those with malignancies were not included in the study.

Our study comprised patients 60 years of age and older who were identified as having T2DM according to the American Diabetes Association criteria [12].

The creatinine-based Chronic Kidney Disease Epidemiology Collaboration (CKD-EPI) equation [13] was used to determine eGFR.

The ethics committee at our hospital granted ethical permission for this investigation under protocol number 4354, dated March 26, 2024.

Every participant provided written informed permission. Our study complies with the principles of the Helsinki Declaration.

To assess the distribution of variables, the Kolmogorov–Smirnov test was performed. The mean ± standard deviation was used to describe parametric data, and the median (interquartile range) was used to express nonparametric data. When necessary, Student's *t*-test, one-way ANOVA, or Kruskal–Wallis test was used to examine group differences. The chi-square test was used to assess categorical variables, which were represented as percentages. Sex-stratified analyses were conducted where feasible; variables that did not meet the minimum expected count of five per cell for the Pearson chi-square test were excluded from sex-specific analyses to preserve statistical validity. Logistic regression analysis (univariate followed by multivariate) was conducted to evaluate the connection between sarcopenia and its components. A *p*-value below 0.05 was considered statistically significant.

## 3. Results

According to our research, 22.6% of individuals who have T2DM (*n* = 65) had sarcopenia. The average duration of diabetes was found to be 15 years, and the average age was 72 years (range: 60–99).

In the sarcopenia group, ischemic heart disease and peripheral artery disease were discovered to be significantly higher among the comorbidities. When comparing the sarcopenia group with the nonsarcopenia group in terms of glucose-lowering treatments (metformin, sulfonylureas, thiazolidinediones, SGLT2 inhibitors, DPP4 inhibitors, insulin, basal + bolus insulin, basal insulin, and premixed insulin), there was no discernible statistically significant change (Table 1).

The sarcopenia group had a statistically significant increased rate of diabetic macrovascular complications and microvascular complications, such as diabetic neuropathy and diabetic nephropathy. Patients with slow walking speed in the 4-m walking test, those identified as frail in the frailty test, and those found to be malnourished in the MNA were statistically significantly higher in the sarcopenia group. Patients who were overweight or obese, according to BMI, were statistically significantly lower in the sarcopenia group. When the GNRI was categorized as < 98 (low) and ≥ 98 (high), in the sarcopenia group, patients with a low GNRI were found to be statistically substantially more (Table 1).

Height, waist circumference, and calf circumference were all shown to be statistically substantially lower in the sarcopenia group than in the nonsarcopenia group. In terms of diabetes duration and age, the sarcopenia group was statistically considerably higher compared to the nonsarcopenia group. Regarding laboratory values, HbA1c, eGFR, albumin, and hemoglobin levels were considerably lower in the group with sarcopenia than in the group without it. Among the laboratory values, urea and CRP levels were statistically considerably higher in the sarcopenia group (Table 1).

To address potential sex-related differences, subgroup analyses were performed separately for male and female participants. In male patients, those with sarcopenia were significantly older and had lower height, calf circumference, hemoglobin, albumin, and GNRI scores, as well as higher CRP levels compared with nonsarcopenic males (Table 2). The prevalence of cerebrovascular disease, peripheral artery disease, and nephropathy was also higher in the sarcopenic group. Among females, sarcopenic patients were older and had lower calf circumference, handgrip strength, albumin levels, and waist circumference compared with their nonsarcopenic counterparts (Table 3). The frequency of retinopathy and normal BMI was higher in sarcopenic females, and GNRI < 98 was significantly more common in this group.

The variables that were significantly correlated with sarcopenia were subjected to multiple logistic regression analysis, as shown in Table 4. First, the Hosmer–Lemeshow test, which is used to assess goodness of fit, showed a *χ*^2^ = 2.222 (*p* = 0.973 > 0.05), indicating that the model is a good fit. The Nagelkerke *R*^2^ value was 0.685, indicating that 68.5% of the variation in sarcopenia status is explained by the variables listed in Table 4.

As a result of the multiple logistic regression analysis, the variables listed in Table 4 were obtained. It was found that the likelihood of having sarcopenia was 4.309 times higher in individuals with macrovascular complications compared to those without. The likelihood of having sarcopenia was 7.326 times higher in individuals with neuropathy, a microvascular complication, compared to those without neuropathy. Additionally, individuals with a slow walking speed in the 4-m walking test were found to be 46.818 times more likely to have sarcopenia in contrast to those with normal walking speed (Table 4).

When comparing patients with a GNRI < 98 (low) and ≥ 98 (high) in terms of comorbidities, a higher prevalence of hypertension was found in the low GNRI group. In the high GNRI group, the prevalence of microvascular complications and neuropathy among the microvascular complications was statistically significantly higher. In the low GNRI group, a statistically significant higher prevalence of frail patients based on frailty scores and malnourished patients based on the MNA was found. When evaluated based on BMI, overweight and obese patients were discovered to be statistically considerably lower in the low GNRI group (Table 5).

When comparing patients with low GNRI to those with high GNRI, waist circumference and calf circumference were discovered to be considerably lower in the low GNRI group. Additionally, the low GNRI group had a statistically significantly higher age. The duration of diabetes diagnosis was discovered to be considerably shorter in the low GNRI group. Among the laboratory values, total cholesterol, LDL cholesterol, eGFR, albumin, and hemoglobin levels were considerably lower in the low GNRI group. In contrast, urea, creatinine, and CRP levels were found to be considerably higher in the low GNRI group (Table 5).

## 4. Discussions

Sarcopenia, often linked to chronic inflammation, insulin resistance, and malnutrition, is a common comorbidity in elderly patients with T2DM. Nutritional indices, such as the GNRI, have been suggested as potential predictors of sarcopenia. Takahashi and Matsuura reported that low GNRI was associated with higher sarcopenia prevalence among elderly T2DM patients, though they did not evaluate specific threshold values [2, 9]. Consistent with these findings, we observed a higher prevalence of low GNRI among sarcopenic elderly diabetic patients. Although we applied the EWGSOP criteria instead of the Asian Working Group criteria, the direction of the association was consistent. While GNRI did not emerge as an independent predictor in multivariate analysis, our subgroup analysis reinforced the clinical relevance of GNRI < 98, a threshold commonly linked to poor nutritional status, frailty, and muscle loss in older adults.

Malnutrition and metabolic dysregulation are proposed contributors to muscle loss in diabetes. Alfaro-Alvarado FA et al. found poor glycemic control and low nutritional status were associated with reduced muscle mass [10]. In line with this, we observed lower BMI and higher prevalence of GNRI < 98 among sarcopenic patients. However, after adjustment for confounders, BMI and GNRI were not independently associated with sarcopenia, highlighting the multifactorial nature of muscle loss, where inflammation and microvascular complications may exert stronger effects.

Previous research has also linked GNRI to frailty and general health outcomes. Zhao Y et al. reported GNRI effectively identifies nutritional risk and frailty in hospitalized elderly patients [11], and Velázquez‐Alva et al. found high sarcopenia prevalence among undernourished diabetic women in nursing homes [14]. These findings support our observation that sarcopenic diabetic individuals frequently exhibit low GNRI, emphasizing the role of nutritional assessment in diabetes care.

The relationship between diabetes and sarcopenia is well established. Wang T et al. demonstrated that older adults with T2DM have a higher risk of sarcopenia and presarcopenia compared to nondiabetic individuals [15]. Although our study did not include a nondiabetic comparison group, the high prevalence of sarcopenia among our participants may reflect the influence of chronic conditions commonly associated with diabetes.

Glycemic control is another critical factor. High HbA1c levels have been associated with increased sarcopenia risk and diabetes-related complications [16, 17]. Interestingly, in our study, sarcopenic patients had lower HbA1c, which may reflect dietary restriction, reduced caloric intake, or comorbidities in advanced age.

Kim K-S et al. reported lower appendicular muscle mass in elderly men with T2DM compared to nondiabetic individuals [18]. Furthermore, both men and women with T2DM have been shown to have reduced muscle mass compared with nondiabetic controls. In our study, although the overall prevalence of sarcopenia did not differ significantly between sexes, subgroup analyses revealed sex-specific clinical patterns. In men, sarcopenia was associated with higher CRP levels, lower albumin and GNRI scores, and a greater prevalence of vascular complications, whereas in women, it was related to lower waist circumference, albumin levels, and handgrip strength, as well as a higher frequency of retinopathy. These findings suggest that the underlying mechanisms and clinical correlates of sarcopenia may differ between men and women with T2DM.

Inflammatory status has been proposed as a key factor linking nutrition and sarcopenia. Gärtner et al. reported correlations between GNRI, inflammatory markers, and hospital stay duration in elderly patients [19]. In line with this, our study revealed higher C-reactive protein levels and lower albumin concentrations among diabetic individuals with low GNRI, further supporting the association between inflammation and poor nutritional state in sarcopenic diabetics.

Treatment-related factors showed no consistent association. While some studies suggest insulin or DPP-4 inhibitors may modulate muscle loss [20, 21], Sazlina et al. found no link between insulin sensitizers and sarcopenia risk [22]. Consistent with this, our findings revealed no significant relationship between oral antidiabetic or insulin therapy and sarcopenia.

Microvascular complications further contribute to sarcopenia. Massimino et al. demonstrated a strong association between diabetic neuropathy and sarcopenia [23], and Pechmann LM et al. reported albuminuria increased sarcopenia likelihood [24]. In our cohort, sarcopenic patients exhibited higher rates of neuropathy, lower eGFR, and higher nephropathy prevalence, reinforcing the link between microvascular complications and muscle loss.

Taken together, these findings indicate that sarcopenia in elderly patients with T2DM arises from a combination of poor nutritional status, inflammation, metabolic dysregulation, and chronic complications. Assessing nutritional status using GNRI may facilitate early identification of high-risk individuals.

Limitations include the cross-sectional design and modest sample size, which preclude causal inference and limit generalizability. Unmeasured factors, such as physical activity, dietary intake, and inflammatory cytokines, could also influence results. Prospective studies are needed to clarify the temporal relationships between GNRI, glycemic control, inflammation, and sarcopenia, and to evaluate whether nutritional interventions can mitigate muscle loss in elderly diabetic patients.

## 5. Conclusion

Based on our findings, elderly patients with T2DM may benefit from evaluation for sarcopenia when screening for microvascular and macrovascular complications. This recommendation was derived from the observed associations in our study rather than a predefined hypothesis. Our results highlight the importance of early detection and, if possible, prevention of sarcopenia in patients with T2DM.

## Figures and Tables

**Table 1:** Comparison of general characteristics and laboratory findings between type 2 diabetic patients with and without sarcopenia.

Variable	Sarcopenia		Test statistics	
	Sarcopenia-*n* (%)	Sarcopenia + *n* (%)	χ^2^	*P*
Gender				
Female	121 (53.3)	32 (49.2)	0.130	0.718
Male	106 (46.7)	33 (50.8)		
Smoking	62 (27.3)	23 (35.4)	1.595	0.207
Alcohol	14 (6.2)	7 (10.8)	1.603	0.205
Hypertension	183 (80.6)	54 (83.1)	0.200	0.655
Hyperlipidemia	68 (30.0)	20 (30.8)	0.016	0.900
Chronic renal disease	52 (22.9)	22 (33.8)	3.196	0.074
COPD^∗^	28 (12.3)	8 (12.3)	0.001	0.995
Cerebrovascular disease	26 (11.5)	10 (15.4)	0.722	0.395
Ischemic heart disease	84 (37.0)	33 (50.8)	3.987	0.046
Peripheral artery disease	20 (8.8)	12 (18.5)	**4.823**	**0.028**
Antidiabetic agents				
Metformin	151 (66.5)	36 (55.4)	2.721	0.099
Sulfonylurea	39 (17.2)	5 (7.7)	3.555	0.059
Thiazolidinedione	8 (3.5)	4 (6.2)	0.887	0.346
SGLT2 inhibitor	39 (17.2)	14 (21.5)	0.646	0.422
DPP-4 inhibitors	93 (41.0)	24 (36.9)	0.344	0.557
Insulin	94 (41.4)	28 (43.1)	0.058	0.810
Basal + bolus insulin	47 (20.7)	14 (21.5)	0.021	0.884
Basal insulin	33 (14.5)	9 (13.8)	0.020	0.889
Premix insulin	16 (7.0)	8 (12.3)	1.853	0.173
Microvascular complication	146 (64.3)	44 (67.7)	0.253	0.615
Macrovascular complication	104 (45.8)	41 (63.1)	**6.023**	**0.014**
Microvascular complication				
Retinopathy	78 (34.4)	30 (46.2)	3.015	0.082
Neuropathy	97 (42.7)	37 (56.9)	**4.099**	**0.043**
Nephropathy	78 (34.4)	32 (49.2)	**4.758**	**0.029**
4-m walk gait speed				
Slow walking speed, < 0.8 m/s	28 (12.3)	58 (89.2)	**143.802**	**< 0.001**
Usual walking speed, ≥ 0.8 m/s	199 (87.7)	7 (10.8)		
Frail Scale				
Robust	98 (43.2)	2 (3.1)	**90.805**	**< 0.001**
Prefrail	95 (41.8)	15 (23.1)		
Frail	34 (15.0)	48 (73.8)		
Mini Nutritional Assessment Scales				
Well-nourished	137 (60.4)	6 (9.2)	**93.379**	**< 0.001**
At risk of malnutrition	74 (32.6)	23 (35.4)		
Malnutrition	16 (7.0)	36 (55.4)		
Body mass index				
Normal weight	33 (14.5)	21 (32.3)	**10.588**	**0.005**
Overweight	114 (50.2)	26 (40.0)		
Obesity	80 (35.2)	18 (27.7)		
GNRI score				
< 98	101 (44.5)	46 (70.8)	**13.956**	**< 0.001**
≥ 98	126 (55.5)	19 (29.2)		
	*Mean ± S.D.*	*Mean ± S.D.*	*T*	*P*
Height, cm	164.7 ± 8.0	161.2 ± 7.3	**2.952**	**0.003**
Waist circumference, cm	101.9 ± 17.3	90.8 ± 19.2	**4.378**	**0.001**
	*Median (min., max.)*	*Median (min., max.)*	*Z*	*P*
Diabetes duration, yr	15.0 (1.0; 40.0)	18.5 (1.0; 50.0)	**2.366**	**0.018**
Age, yr	70.0 (60.0; 90.0)	75.0 (60.0; 99.0)	**4.617**	**< 0.001**
Calf circumference, cm	39.0 (19.0; 55.0)	25.0 (11.0; 37.0)	**−11.953**	**< 0.001**
Handgrip strength, kg	25.7 (1.2; 38.5)	8.1 (1.3; 22.7)	**−11.479**	**< 0.001**
FPG^∗∗^, mg/dL	145.0 (48.0; 550.0)	136.0 (71.0; 647.0)	−0.826	0.409
Total cholesterol, mg/dL	159.0 (60.0; 463.0)	152.5 (88.0; 290.0)	−1.061	0.289
LDL cholesterol, mg/dL	85.0 (20.0; 251.0)	78.5 (16.0; 198.0)	−1.346	0.178
HbA1C, %	8.0 (6.5; 19.1)	7.2 (6.5; 15.8)	**−3.034**	**0.002**
Urea, mg/dL	45.0 (11.0; 239.0)	60.0 (15.0; 195.0)	**2.508**	**0.012**
Creatinine, mg/dL	0.9 (0.5; 3.1)	1.2 (0.3; 4.7)	1.874	0.061
eGFR^∗∗∗^, mL/min	71.9 (20.2; 159.9)	59.0 (13.5; 229.3)	**−2.280**	**0.023**
CRP^∗∗∗∗^, mg/dL	5.6 (0.0; 13.0)	6.0 (0.0; 17.0)	1.778	0.075
Albumin, g/L	38.1 (22.9; 49.6)	34.7 (2.6; 47.7)	**−3.699**	**< 0.001**
Hemoglobin, g/dL	11.6 (4.2; 16.7)	10.0 (6.4; 14.8)	**−3.985**	**< 0.001**

*Note:* The bold values denote statistically significant results (*p* < 0.05).

^∗^Chronic obstructive pulmonary disease.

^∗∗^Fast plasma glucose.

^∗∗∗^Estimated glomerular filtration rate.

^∗∗∗∗^C-reactive protein.

**Table 2:** Comparison of general characteristics and laboratory findings between type 2 diabetic male patients with and without sarcopenia.

Variable	Sarcopenia		Test statistics	
	Sarcopenia-*n* (%)	Sarcopenia + *n* (%)	χ^2^	*P*
Smoking	50 (47.2)	16 (50.0)	0.079	0.779
Alcohol	11 (10.4)	6 (18.8)	1.595	0.207
Hypertension	81 (76.4)	23 (71.9)	0.273	0.601
Hyperlipidemia	23 (21.7)	9 (28.1)	0.570	0.450
Chronic renal disease	29 (27.4)	13 (40.6)	2.043	0.153
COPD^∗^	19 (17.9)	5 (15.6)	0.090	0.764
Cerebrovascular disease	8 (7.5)	7 (21.9)	**5.208**	**0.022**
Ischemic heart disease	47 (44.3)	18 (56.3)	1.399	0.237
Peripheral artery disease	14 (13.2)	9 (28.1)	**3.938**	**0.047**
Antidiabetic agents				
Metformin	70 (66.0)	16 (50.0)	2.692	0.101
SGLT2 inhibitor	21 (19.8)	8 (25.0)	0.399	0.528
DPP-4 inhibitors	43 (40.6)	15 (46.9)	0.402	0.526
Insulin	45 (42.5)	11 (34.4)	0.665	0.415
Basal + bolus insulin	24 (22.6)	5 (15.6)	0.729	0.393
Basal insulin	14 (13.2)	5 (15.6)	0.121	0.728
Microvascular complication	67 (63.2)	20 (62.5)	0.005	0.942
Macrovascular complication	55 (51.9)	22 (68.8)	2.834	0.092
Microvascular complication				
Retinopathy	39 (36.8)	13 (40.6)	0.154	0.695
Neuropathy	42 (39.6)	17 (53.1)	1.831	0.176
Nephropathy	36 (34.0)	18 (56.3)	**5.126**	**0.024**
Normal weight	15 (14.2)	8 (25.0)	2.108	0.349
Overweight	51 (48.1)	13 (40.6)		
Obesity	40 (37.7)	11 (34.4)		
GNRI score				
< 98	48 (45.3)	21 (65.6)	**4.068**	**0.044**
≥ 98	58 (54.7)	11 (34.4)		
	*Mean ± S.D.*	*Mean ± S.D.*	*T*	*P*
Height, cm	164.0 ± 8.3	159.3 ± 6.6	**2.648**	**0.009**
Calf circumference, cm	39.1 ± 5.3	24.7 ± 4.4	**13.424**	**< 0.001**
Albumin	38.0 ± 5.1	35.4 ± 4.8	**2.063**	**0.041**
Hemoglobin	11.6 ± 2.1	10.1 ± 1.6	**3.716**	**< 0.001**
	*Median (min., max.)*	*Median (min., max.)*	*Z*	*P*
Diabetes duration, yr	15.0 (1.0; 40.0)	22.0 (1.0; 35.0)	1.775	0.076
Age, yr	70.0 (60.0; 90.0)	76.0 (62.0; 99.0)	**3.801**	**< 0.001**
Waist circumference, cm	102.5 (68.0; 139.0)	98 (59.0; 130.0)	−1.678	0.093
Handgrip strength, kg	25.5 (1.2; 37.0)	8.5 (1.3; 22.7)	**−7.676**	**< 0.001**
FPG^∗∗^, mg/dL	149.0 (65.0; 550.0)	115.0 (78.0; 283.0)	**−2.056**	**0.040**
Total cholesterol, mg/dL	169.0 (73.0; 463.0)	171.0 (88.0; 272.0)	−1.367	0.171
LDL cholesterol, mg/dL	96.0 (28.0; 251.0)	76.0 (20.0; 144.0)	−1.887	0.059
HbA1C, %	8.1 (6.5; 19.1)	6.9 (6.5; 11.4)	**−3.830**	**< 0.001**
Urea, mg/dL	48.0 (16.0; 208.0)	61.0 (15.0; 169.0)	1.430	0.153
Creatinine, mg/dL	1.0 (0.5; 3.0)	1.1 (0.4; 4.7)	1.350	0.177
eGFR^∗∗∗^, mL/min	68.4 (22.2; 131.9)	58.3 (13.5; 209.2)	−1.816	0.069
CRP^∗∗∗∗^, mg/dL	5.0 (0.0; 12.1)	7.0 (0.0; 17.0)	**3.053**	**0.002**

*Note:* The bold values denote statistically significant results (*p* < 0.05).

^∗^Chronic obstructive pulmonary disease.

^∗∗^Fast plasma glucose.

^∗∗∗^Estimated glomerular filtration rate.

^∗∗∗∗^C-reactive protein.

**Table 3:** Comparison of general characteristics and laboratory findings between type 2 diabetic female patients with and without sarcopenia.

Variable	Sarcopenia		Test statistics	
	Sarcopenia-*n* (%)	Sarcopenia + *n* (%)	*χ* ^2^	*P*
Smoking	12 (9.9)	7 (21.2)	3.058	0.080
Hyperlipidemia	45 (37.2)	11 (33.3)	0.167	0.683
Chronic renal disease	23 (19.0)	9 (27.3)	1.076	0.300
Ischemic heart disease	37 (30.6)	15 (45.5)	2.566	0.109
Antidiabetic agents				
Metformin	81 (66.9)	20 (60.6)	0.461	0.497
SGLT2 inhibitor	18 (14.9)	6 (18.2)	0.215	0.643
DPP-4 inhibitors	50 (41.3)	9 (27.3)	2.166	0.141
Insulin	49 (40.5)	17 (51.5)	1.286	0.257
Basal + bolus insulin	23 (19.0)	9 (27.3)	1.076	0.300
Premix insulin	8 (6.6)	6 (18.2)	**4.200**	**0.040**
Microvascular complication	79 (65.3)	24 (72.7)	0.648	0.421
Macrovascular complication	49 (40.5)	19 (57.6)	3.067	0.080
Microvascular complication				
Retinopathy	39 (32.2)	17 (51.5)	**4.167**	**0.041**
Neuropathy	55 (45.5)	20 (60.6)	2.383	0.123
Nephropathy	42 (34.7)	14 (42.4)	0.667	0.414
Body mass index				
Normal weight	18 (14.9)	13 (39.4)	**9.779**	**0.008**
Overweight	63 (52.1)	13 (39.4)		
Obesity	40 (33.0)	7 (21.2)		
GNRI score				
< 98	53 (43.8)	25 (75.8)	**10.593**	**0.001**
≥ 98	68 (56.2)	8 (24.2)		
	*Mean ± S.D.*	*Mean ± S.D.*	*T*	*P*
Height, cm	165.3 ± 7.8	163.0 ± 7.5	1.483	0.140
Waist circumference, cm	102.3 ± 17.6	87.2 ± 16.9	**4.284**	**< 0.001**
Hemoglobin	11.4 ± 2.4	10.5 ± 2.2	**2.025**	**0.045**
	*Median (min., max.)*	*Median (min., max.)*	*Z*	*P*
Diabetes duration, yr	14.0 (1.0; 40.0)	16.0 (1.0; 50.0)	1.527	0.127
Age, yr	70.0 (60.0; 89.0)	75.0 (62.0; 96.0)	**2.733**	**0.006**
Calf circumference, cm	39.0 (19.0; 55.0)	25.0 (11.0; 30.0)	**−8.670**	**< 0.001**
Handgrip strength, kg	26.8 (3.9; 38.5)	7.0 (2.0; 15.6)	**−8.492**	**< 0.001**
FPG∗∗, mg/dL	139.0 (48.0; 394.0)	144.0 (71.0; 647.0)	0.828	0.408
Total cholesterol, mg/dL	152.0 (60.0; 317.0)	146.0 (89.0; 290.0)	−0.161	0.872
LDL cholesterol, mg/dL	77.0 (20.0; 215.0)	81.0 (16.0; 198.0)	−0.018	0.986
HbA1C, %	7.8 (6.5; 17.2)	7.4 (6.5; 15.8)	−0.609	0.542
Urea, mg/dL	40.0 (11.0; 239.0)	59.0 (17.0; 195.0)	**2.030**	**0.042**
Creatinine, mg/dL	0.9 (0.5; 3.1)	1.2 (0.3; 3.2)	1.398	0.162
eGFR∗∗∗, mL/min	76.2 (20.2; 159.9)	59.1 (20.9; 229.3)	−1.429	0.153
CRP∗∗∗∗, mg/dL	6.0 (0.0; 13.0)	6.0 (0.0; 12.1)	−0.179	0.858
Albumin	37.6 (23.6; 49.6)	34.6 (2.6; 47.7)	**−2.977**	**0.003**

*Note:* The bold values denote statistically significant results (*p* < 0.05).

^∗∗^Fast plasma glucose.

^∗∗∗^Estimated glomerular filtration rate.

^∗∗∗∗^C-reactive protein.

**Table 4:** Results of the multiple logistic regression model for variables that related to sarcopenia.

Variable	β^	S.E.β^	Wald	*P*	Exp (β)	95% C.I odds ratio	
						Lower	Upper
Constant	−5.215	1.077	23.442	< 0.001	0.005		
Ischemic heart disease	−0.684	0.646	1.122	0.289	0.504	0.142	1.789
Peripheral artery disease	−0.879	0.714	1.517	0.218	0.415	0.102	1.682
Macrovascular complication	1.461	0.704	4.307	0.038	4.309	1.085	17.119
Neuropathy	1.991	0.651	9.365	0.002	7.326	2.046	26.322
Nephropathy	−0.025	0.568	0.002	0.965	0.975	0.321	2.966
Slow walking speed	3.846	0.712	29.164	< 0.001	46.818	11.592	189.081
Prefrail	1.392	0.947	2.162	0.141	4.024	0.629	25.749
Frail	1.502	1.028	2.135	0.144	4.491	0.599	33.672
At risk of malnutrition	−0.374	0.799	0.219	0.640	0.688	0.144	3.296
Malnutrition	1.156	0.957	1.459	0.227	3.176	0.487	20.713
Overweight	−0.964	0.589	2.679	0.102	0.382	0.120	1.210
Obesity	−0.506	0.654	0.597	0.440	0.603	0.167	2.175
GNRI score ≥ 98	−0.563	0.512	1.209	0.272	0.570	0.209	1.553

*Note:* −2 Log likelihood = 136.400.

**Table 5:** Comparison of general characteristics and laboratory findings in GNRI subgroups of patients with type 2 diabetes.

Variable	GNRI score		Test statistics	
	< 98n (%)	≥ 98n (%)	*T*	*P*
Gender				
Female	78 (53.1)	76 (52.4)	0.012	0.912
Male	69 (46.9)	69 (47.6)		
Smoking	42 (28.6)	43 (29.7)	0.042	0.838
Alcohol	11 (7.5)	10 (6.9)	0.038	0.846
Hypertension	127 (86.4)	110 (75.9)	**5.297**	**0.021**
Hyperlipidemia	43 (29.3)	45 (31.0)	0.110	0.740
Chronic renal disease	39 (26.5)	35 (24.1)	0.221	0.638
COPD^∗^	20 (13.6)	16 (11.0)	0.446	0.504
Cerebrovascular disease	15 (10.2)	21 (14.5)	1.236	0.266
Ischemic heart disease	66 (44.9)	51 (35.2)	2.875	0.090
Peripheral artery disease	13 (8.8)	19 (13.1)	1.358	0.244
Antidiabetic agents				
Metformin	89 (60.5)	98 (67.6)	1.572	0.210
Sulfonylurea	21 (14.3)	23 (15.9)	0.142	0.707
SGLT2 inhibitors	21 (14.3)	32 (22.1)	2.977	0.084
DPP-4 inhibitors	56 (38.1)	61 (42.1)	0.480	0.488
Insulin	64 (43.5)	58 (40.0)	0.376	0.540
Basal + bolus insulin	33 (22.4)	28 (19.3)	0.435	0.509
Basal insulin	23 (15.6)	19 (13.1)	0.383	0.536
Premix insulin	12 (8.2)	12 (8.3)	0.001	0.972
Microvascular complication	79 (53.7)	111 (67.7)	**16.710**	**< 0.001**
Macrovascular complication	71 (48.3)	74 (51.0)	0.218	0.640
Microvascular complication				
Retinopathy	47 (32.0)	61 (42.1)	3.193	0.074
Neuropathy	56 (38.1)	78 (53.8)	**7.244**	**0.007**
Nephropathy	54 (36.7)	56 (38.6)	0.111	0.739
4-m walk gait speed				
Slow walking speed,< 0.8 m/s	60 (40.8)	26 (17.9)	**18.400**	**< 0.001**
Usual walking speed, ≥ 0.8 m/s	87 (59.2)	119 (82.1)		
Frail Scale				
Robust	39 (26.5)	61 (42.1)	**21.941**	**< 0.001**
Prefrail	49 (33.3)	61 (42.1)		
Frail	59 (40.2)	23 (15.8)		
Mini Nutritional Assessment Scales				
Well-nourished	53 (36.1)	90 (62.1)	**27.704**	**< 0.001**
At risk of malnutrition	53 (36.1)	44 (30.3)		
Malnutrition	41 (27.8)	11 (7.6)		
Body mass index				
Normal weight	37 (25.2)	17 (11.7)	**10.035**	**0.007**
Overweight	69 (46.9)	71 (49.0)		
Obesity	41 (27.9)	57 (39.3)		
	*Mean ± S.D*	*Mean ± S.D.*	*T*	*P*
Height, cm	164.7 ± 7.5	163.1 ± 8.4	1.425	0.155
Waist circumference, cm	95.3 ± 18.4	103.5 ± 17.4	**−3.599**	**< 0.001**
Hemoglobin, g/dL	10.5 ± 2.1	12.0 ± 2.1	**−6.201**	**< 0.001**
	*Median (min., max.)*	*Median (min., max.)*	*Z*	*P*
Diabetes duration, yr	12.0 (1.0; 50.0)	16.0 (1.0; 40.0)	**2.119**	**0.034**
Age, yr	72.0 (60.0; 94.0)	70.0 (60.0; 99.0)	**−2.688**	**0.007**
Calf circumference, cm	35.0 (11.0; 54.0)	39.0 (16.0; 55.0)	**4.908**	**< 0.001**
Handgrip strength, kg	21.3 (1.2; 38.5)	24.1 (2.0; 36.5)	1.490	0.136
FPG∗∗, mg/dL	145.0 (48.0; 647.0)	141.0 (66.0; 476.0)	0.613	0.540
Total cholesterol, mg/dL	140.5 (60.0; 290.0)	174.0 (84.0; 463.0)	**4.409**	**< 0.001**
LDL cholesterol, mg/dL	75.0 (16.0; 198.0)	94.0 (28.0; 251.0)	**3.250**	**0.001**
HbA1C, %	7.6 (6.5; 19.1)	7.8 (6.5; 17.0)	1.200	0.230
Urea, mg/dL	55.0 (11.0; 239.0)	40.0 (16.0; 137.0)	**−3.187**	**0.001**
Creatinine, mg/dL	1.1 (0.3; 4.7)	0.9 (0.5; 2.4)	**−2.647**	**0.008**
eGFR^∗∗∗^, mL/min	62.3 (13.5; 229.3)	76.9 (20.6; 133.3)	**2.121**	**0.034**
CRP^∗∗∗∗^, mg/dL	6.0 (1.0; 17.0)	5.0 (0.0; 13.0)	**−3.488**	**< 0.001**
Albumin, g/L	33.3 (2.6; 41.3)	41.2 (37.5; 49.6)	**14.647**	**< 0.001**

*Note:* The bold values indicate statistically significant results (*p* < 0.05) in the comparison between the GNRI subgroups.

^∗^Chronic obstructive pulmonary disease.

^∗∗^Fast plasma glucose.

^∗∗∗^Estimated glomerular filtration rate.

^∗∗∗∗^C-reactive protein.

## Data Availability

The data that support the findings of this study are available from the corresponding author upon reasonable request.
